# Peer effects on risk behaviour: the importance of group identity

**DOI:** 10.1007/s10683-016-9478-z

**Published:** 2016-03-18

**Authors:** Francesca Gioia

**Affiliations:** School of Economics, University of Edinburgh, 30 Buccleuch Place, Edinburgh, EH8 9JT UK

**Keywords:** Peer effects, Group identity, Risk behaviour, Ranking, D03, D81, D83, Z13

## Abstract

This paper investigates whether and to what extent group identity plays a role in peer effects on risk behaviour. We run a laboratory experiment in which different levels of group identity are induced through different matching protocols (random or based on individual painting preferences) and the possibility to interact with group members via an online chat in a group task. Risk behaviour is measured by using the Bomb Risk Elicitation Task and peer influence is introduced by giving subjects feedback regarding group members’ previous decisions. We find that subjects are affected by their peers when taking decisions and that group identity influences the magnitude of peer effects: painting preferences matching significantly reduces the heterogeneity in risk behaviour compared with random matching. On the other hand, introducing a group task has no significant effect on behaviour, possibly because interaction does not always contribute to enhancing group identity. Finally, relative riskiness within the group matters and individuals whose peers are riskier than they are take on average riskier decisions, even when controlling for regression to the mean.

**Electronic supplementary material:**

The online version of this article (doi:10.1007/s10683-016-9478-z) contains supplementary material, which is available to authorized users.

## Introduction

The question of whether and how peers influence an individual’s behaviour has been widely investigated in economics literature. Considerable evidence suggests that individuals who are physically or socially close to a subject influence his/her behaviour and choices. Peers’ influence has been studied in the context of academic achievement, choice of university degree course, worker productivity, cheating behaviour and social outcomes such as joining student societies (Manski 1993; Sacerdote 2001; Zimmerman 2003; Stinebrickner and Stinebrickner 2006; Falk and Ichino 2006; Carrell et al. 2008; Mas and Moretti 2009; Imberman et al. 2012; Falk et al. 2013).

Peer effects have also often been mentioned as a leading explanation for why people engage in risk taking activities such as smoking (Alexander et al. 2001), drug and alcohol use (Fergusson et al. 2002; Duncan et al. 2005; Powell et al. 2005; Lundborg 2006; Clark and Lohéac 2007), criminal activity (Fergusson et al. 2002; Bayer et al. 2009), financial decisions (Kelly and O’Grada 2000; Hong et al. 2004; Brown et al. 2008; Bursztyn et al. 2014; Cai et al. 2015) and entrepreneurship decisions (Nanda and Sørensen 2010; Falck et al. 2012; Lerner and Malmendier 2013).

Despite their relevance for many social and economic interactions, little is known about the circumstances triggering peer effects. In this paper, we investigate the role of group identity, which psychologists define as “the portion of an individual’s self-concept derived from the sense of belonging to the social group” (Hogg and Vaughan 2002). Group membership is a ubiquitous feature of social and economic life. However, groups vary enormously and so does people’s attachment to different social groups. We hypothesize that the sense of belonging to a social group may affect the realization and magnitude of peer effects.

Since the introduction of the minimal group paradigm by Tajfel (1970) and the subsequent development of the social identity theory (Billig and Tajfel 1973), different levels of group identity have been introduced to understand how and why people behave differently towards those that they share a common identity with. In particular, numerous studies document that people tend to behave more prosocially when they interact with members of their own group, but become less generous, less trusting, and less cooperative towards individuals who belong to different groups (Tajfel et al. 1971; Götte et al. 2006; Charness et al. 2007; Chen and Li 2009).

The goal of this paper is to study whether and to what extent group identity plays a role in peer effects on risk behaviour. Some recent evidence suggests that not all peers matter and some matter more than others (Vaquera and Kao 2008; Lomi et al. 2011; Lin and Weinberg 2014; Borjas and Doran 2015). The sense of belonging to a group may be a possible explanation for this finding: individuals may only be affected by social groups they feel they belong to and the peers they are particularly attached to may matter more than other peers. Knowing that group identity is one of the mechanisms triggering peer effects may help the design of policy interventions the benefits of which may be increased by choosing the target peer group wisely. Also, considering that risk is at play in a large range of social economic decisions, such as choice of career, university degree course or study effort (Saks and Shore 2005; Belzil and Leonardi 2007; Caner and Okten 2010; De Paola and Gioia 2012), and that recent evidence shows that an individual’s risk behaviour is shaped by the behaviour of others in the immediate social environment, studying the role that group identity has in an individual’s decision-making when faced with risk would appear to be especially worthwhile. To our knowledge, we are the first to study how the degree of group identity interacts with peer effects.

In this paper, we use procedures commonly used in the literature to induce different levels of group identity (Tajfel 1970; Chen and Li 2009) with the aim of investigating the impact of group identity on the magnitude of peer effects on an individual’s decisions in a risky setting.

We run a laboratory experiment with 255 students. We measure individual risk behaviour by using the Bomb Risk Elicitation Task, an easy task in which subjects have to choose how many boxes to collect out of 100, 99 of which contain £0.10 while one contains a bomb. Earnings increase linearly with the number of boxes collected, but are zero if the bomb is collected. Peer influence is introduced by providing subjects with feedback on fellow group members’ decisions in the immediately preceding performance of the task.

The experiment consists of a control group and four treatments. One treatment, called *Anchoring* treatment, is meant to distinguish peer effects from anchoring effects that may arise if the change in individual behaviour is driven by the exposure to numbers rather than by a desire to be similar to assigned peers. The other three treatments introduce a different level of group identity. The *Random* treatment matches individuals into groups of three at random. The *Painting* treatment introduces a less impersonal matching: individuals are first asked to express their painting preferences, by choosing their favourite paintings from within five pairs of paintings, and then are matched according to their painting preferences. Finally, the *Chat* treatment matches individuals according to their painting preferences and entails a group task which consists of their guessing the name of the artists responsible for two more paintings by using an online chat to ask for help from and offer aid to their fellow group members. This additional task is meant to enhance the level of perceived group identity by letting people interact with fellow group members more.

We find evidence of peer effects in risk behaviour and find that they depend on the level of group identity. Individuals who are assigned to groups based on their painting preferences are more likely to conform to their peers than the control group (the group standard deviation falls by 8.5 boxes collected in the BRET) and the anchoring treatment (−7.8 boxes). Also, enhancing the level of group identity, by making people aware that they have the same painting preferences as their peers, significantly increases (by about 4.4 boxes) peer effects beyond those produced through a random group assignment.

The chat treatment, which combines a preferences-based matching with a group task, does not induce significantly different peer effects from those found for the painting treatment (−7.8 boxes). We speculate that this may be because the group task has a different effect on perceived group identity as a consequence of the individual experience in the task. Indeed, we find that when interaction in the group task contributes to the enhancing of group identity, the magnitude of peer effects on risk behaviour does increase in comparison with the painting treatment. For example, with regard the control group, groups in the chat treatment whose participants consider their group to be more helpful than the average significantly reduce their heterogeneity in risk behaviour by 5.2 boxes more than groups in the chat treatment who do not find their group very helpful, and by 3.8 boxes more than groups in the painting treatment. Similar results are found for groups whose participants feel more attached than the average to their peers or reach an agreement on the possible answers in the group task very quickly.

The relative position of the individual within the group in terms of risk behaviour plays an important role in the individual’s decisions when receiving feedback about peers’ previous decisions. Individuals whose peers are riskier than they are tend to increase their choice by 12.3 boxes compared with individuals with mixed peers, while individuals whose peers are less risky than they are tend to decrease it on average by 5.5 boxes. When ruling out the component of the effect due to regression to the mean, peers’ risk behaviour continues to play a significant role for bottom ranked individuals (+6.9 boxes, significant at the 1 % level) while the effect is very close to zero for top ranked individuals.

The paper is structured in five parts. Section 2 presents a brief overview of the related literature. In Sect. 3, we describe our experimental design. Section 4 presents our empirical analysis. Section 5 concludes.

## Literature review

This paper combines three different branches of economics and psychology literature: research investigating the extent to which an individual’s behaviour is modified by his/her peers; research looking at the determinants of risk behaviour; research into the development of a group identity and its effects. Only a few very recent papers integrate the literature on peer effects and the literature on risk attitudes to look at the role played by peers in an individual’s risk behaviour, but no one induces different levels of group affiliation as we did.

Gardner and Steinberg (2005) investigate the impact of peers on the orientation towards risk of different age groups and find that, on average, individuals are more risk seeking when in the company of their peers than when alone and that peer influence plays a stronger role in explaining risky behaviour among adolescents and youths than it does among adults. Unlike our study, the authors do not use incentivized tasks, but pay a fixed fee and, instead of giving feedback on peers’ choices, they let peers work together or intervene when other peers are working. More importantly, they investigate the emergence of peer effects in a setting with a very high level of group identity because they require participants to invite two people they know of the same gender to the session and let these three people constitute a peer group.

Cooper and Rege (2011) show the existence of peer group effects in a series of binary choices under risk and ambiguity by using feedback about the choices made by other subjects as the channel for peer influence. They find that peer effects in risk behaviour may be explained by social regret, that is an individual decides to behave similarly to his/her peers because s/he experiences a lower loss in utility from not taking an action that would have led to higher payoffs ex post if his/her peers have also not taken that action. In our experiment, social regret is ruled out by design as a possible cause of peer effects because participants never choose from among the same lotteries. Thus even when choosing the same action, they might end up with different payoffs. Moreover, participants have all the time they need to make their decision in our setting, while time pressure might play a role in Cooper and Rege’s experiments because, if subjects do not take a choice within about 1 min, it is randomly taken by the computer.^1^

Another experimental paper investigating peer effects on risk behaviour is by Lahno and Serra-Garcia (2015). They use binary lottery choices as a task to be performed to test for two causes of peer effects, utility from payoff differences and utility from conforming to peers, and show that peer effects are mainly explained by the former and that responses to peers’ decisions depend on whether peers’ choices are voluntary or randomly imposed by the experimenter. As in our experiment, they use feedback on the peers’ decision as the channel for peer influence; however, while we rule out relative payoff concerns by design, their focus is on the direct impact of payoff differences.

Evidence of peer effects on risk behaviour is also found by Bougheas et al. (2013), who use a laboratory experiment to study the importance of two channels, consultation and feedback, for peer interaction, rather than the causes of peer effects, and by Balsa et al. (2015) and Ahern et al. (2013), who use survey data on adolescents and MBA students, respectively. Finally, Trautmann and Vieider (2012) present an overview of social influences on economic decisions under risk.

The other strand of literature this paper refers to, is the literature on group identity. There are two main experimental methods used to study social identity in social psychology: priming natural social identities and artificially inducing group identities. We decided in favour of induced group identities because our aim is to look at the effect of an increase in perceived social identity on peer group influence effects on risk behaviour. Priming would make it difficult both to create increasingly stronger group identities and to separate the effect of a stronger group identity per se from the meaning attached to the primed identity.^2^

The literature which induces different levels of group identity has typically used the minimal group paradigm, that is it has categorized people into some groups according to some trivial criterion, such as visual judgements (estimating the number of dots flashed onto a screen) or painting preferences (choosing between Klee or Kandinsky paintings), and has further increased the saliency of the group by introducing payoff communality and interaction among group members (Charness et al. 2007; Chen and Li 2009; Güth et al. 2009; Sutter 2009; Arora et al. 2012).

This literature has mainly studied the role of social categorization in inter-group discrimination and social preferences and has shown that individuals who are assigned to novel social categories discriminate in favour of their own category. There are no studies that focus on the role of risk behaviour and use a setting where the individual decides just for himself and not for the other participants.

Among the main papers which study the effect of inducing a greater sense of group identity, Chen and Li (2009) look at social preferences and find that individuals are both more charitable and less envious towards members of the same group than towards people from outside the group and both more likely to reward a fellow group member *vs* an outsider for good behaviour and less likely to punish him/her for misbehaviour. Moreover, social welfare maximizing decisions are more likely when subjects are matched with fellow group members. Charness et al. (2007) show that participants act more aggressively to the benefit of their group and at the expense of outsiders as identity becomes more salient. Similarly, Arora et al. (2012) find that increases in group affiliation are accompanied by higher levels of cooperation, personal satisfaction and trust in one’s group.

## Experimental design

The individual level of risk aversion is measured by using the Bomb Risk Elicitation Task—BRET (Crosetto and Filippin 2013). This method measures risk behaviour by having subjects choose how many boxes to collect out of 100, 99 of which contain £0.10 while one contains a bomb.

In both Economics and Psychology, there are a variety of experimental methods for eliciting and assessing risk behaviour (see Charness et al. 2013, for a review of advantages and disadvantages of the most common risk elicitation methods). We use the BRET because of a number of appealing features. First, its duration is very short and it can even be run with paper and pencil, which would allow the repetition of our experiment in the field where access to a computer is limited. This could prove very interesting given that field work would allow observation of levels of group identity that are very close to real-life ones. Second, the BRET is very easy to understand thanks to the visual representation of the game which illustrates probabilities and outcomes intuitively and transparently. Simple methods are most useful in studies like ours which try to capture treatment effects and differences in individual risk preferences (Charness et al. 2013). Moreover, the absence of complexity from the task should reduce the extent to which social learning drives peer effects on individual risk behaviour within our setting.^3^ Finally, compared with other well-known tasks in the literature, the BRET allows precise measurement of both risk aversion and risk seeking, is defined entirely in the gain domain and does not provide any endogenous reference point, thus avoiding the presence of loss aversion as a potential confounding factor.^4^

We induce different levels of group identity following a procedure, similar to the one used by Chen and Li (2009) to study social preferences, that combines two assignment methods (random and based on painting preferences) and a collective problem solving task using an online chat program to enhance feelings of belonging to the assigned group.^5^

### Procedure

We conducted the experiment in April 2014 through computers at the Behavioural Laboratory at the University of Edinburgh (BLUE) and programmed it by using z-Tree (Fischbacher 2007). The experiment consists of four treatments and a control. We ran three sessions for each of the treatments and for the control. Each session was divided into three parts and the treatment protocol (group matching and feedback on group decisions) was introduced for the second and third parts.

Participants were recruited using the ORSEE software (Greiner 2015). A total of 255 students participated in the experiment, distributed over 15 sessions of about 35–40 min each.

Upon entering the laboratory, we randomly assigned participants to a computer. Then, we read aloud the introductory instructions to the experiment, which were also displayed on the participants’ computer screens. We gave detailed instructions at the beginning of each part and, when needed, before each relevant step in the experiment. On each occasion, after reading the instructions, we gave individuals some time to ask clarifying questions.

Participants always had to perform the same task: the Bomb Risk Elicitation Task (BRET). In both Parts I and II of the experiment, participants performed the BRET once. They performed the BRET 10 times in Part III of the experiment.

**Fig. 1:**
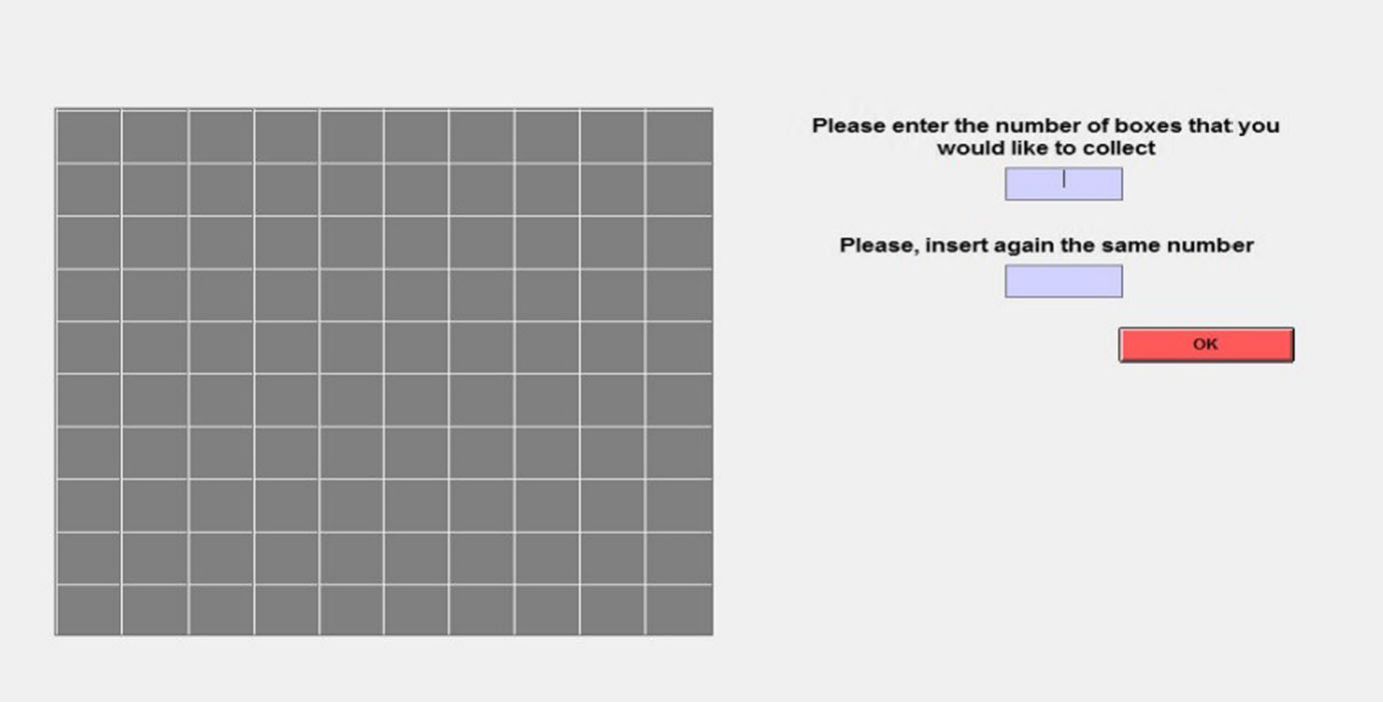
Participant’s computer screen when performing the BRET

When playing the BRET, subjects see a square on their PC screen formed of 10 × 10 cells which represent the 100 boxes that they can collect (see Fig. 1). They have to choose how many boxes to collect and write down their chosen number.^6^ They can therefore choose their preferred lottery among 100 lotteries whose outcomes and probabilities are fully described by just one parameter, i.e. the number of collected boxes.

Earnings increase linearly with the number of boxes collected, but participants are warned that their earnings are provisional. In fact, they know that one box contains a bomb without knowing which box this is.

Boxes are collected in numerical order starting from number 1 in the top left hand corner and continuing until the number of boxes chosen by the subject is reached. While reading the instructions, we display a dynamic visual representation of the game on the main screen to show the order of collection.

**Fig. 2:**
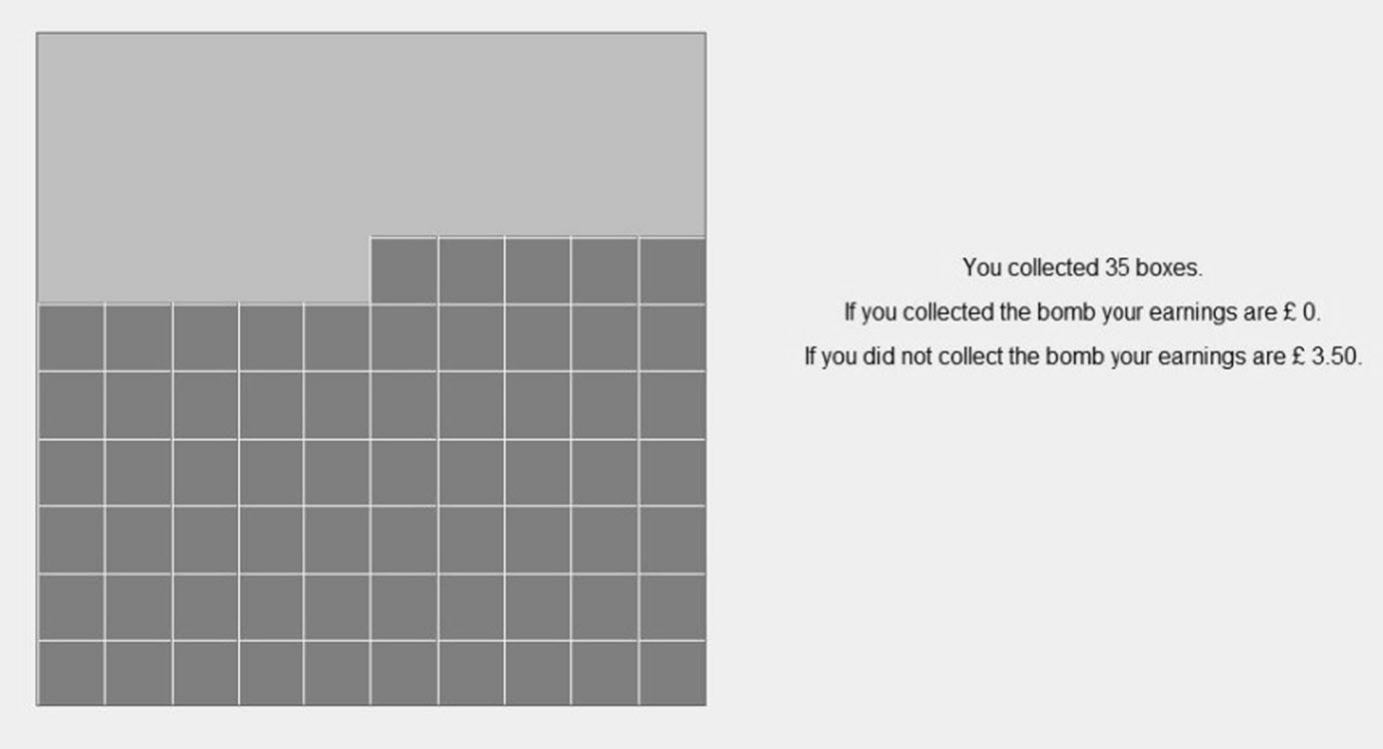
Computer screen for a participant who chose to collect 35 boxes

If a participant collects the bomb, s/he earns zero. If s/he collects a number of boxes inferior to the number of the box containing the bomb (i.e. s/he does not collect the bomb), s/he obtains £0.10 for each collected box. After confirming their decision, participants see the square of boxes on their screen. This shows the collected boxes in light grey and a message with the potential earnings in both situations (if the bomb is collected or not). Figure 2 shows the computer screen for a participant who chose to collect 35 boxes.^7^

Participants are allowed to play a practice round before the beginning of the experiment. This practice round gives them an opportunity to make sure they understand the rules, the types of decisions they will make and how these will affect their earnings. The trial period, however, does not end with the draw of the bomb’s position so as to avoid providing subjects with a reference point regarding the bomb’s position.

At the end of the experiment, participants completed a short questionnaire. Then, one of their decisions and the position of the bomb were selected by separate random draws carried out at the individual level.^8^ The selected decision and the corresponding earnings were shown on the computer screen together with the selected bomb position. We paid out total earnings (including a show-up fee of £3) in cash at the end of the experiment. We called participants individually on the basis of their computer number and they went into another room, signed a receipt and received their earnings in an envelope. Average earnings for participant were £5.45 (including the show-up fee).

### Treatments

We exogenously sorted the experiment participants into a control group, a treatment group (Anchoring) designed to distinguish between anchoring effects^9^ and peer effects^10^ and three treatment groups (Random, Painting, Chat) designed to increase group identity.

Table 1 describes the main features of our treatments. There are 51 participants in the control group; 48 in the random and chat treatments and 54 in the anchoring and painting treatments. Participants in the random treatment are randomly matched into groups of three.

**Table 1:** Treatments of the experiment

Treatments	Group assignment			Information on peers’ previous choices				No. of sessions	No. of subjects
	Random	Preference based	Group task	Part I	Part II	Part III	Channel		
Control	No	No	No	No	No	No	–	3	51
Anchoring	No	No	No	No	Yes	Yes	Indirect	3	54
Random	Yes	No	No	No	Yes	Yes	Direct	3	48
Painting	No	Yes	No	No	Yes	Yes	Direct	3	54
Chat	No	Yes	Yes	No	Yes	Yes	Direct	3	48

In the painting treatment, the matching is based on individual preferences: individuals are shown five pairs of paintings and have to choose their favourite painting within each pair. In each pair, one painting is by Klee and one is by Kandinsky (individuals are not told who the artists are).^11^ After having chosen their favourite paintings, they are assigned to groups of three and receive information about the painting preferences of all the members of their group: an individual prefers Klee to Kandinsky if in at least three out of the five pairs s/he chooses Klee rather than Kandinsky, and vice versa.

The chat treatment is very similar to the painting treatment. The only difference is that, after being matched into groups of three based on their painting preferences and before performing the BRET again with information, individuals in the chat treatment have to perform a group task. Subjects are shown two additional paintings^12^ and are given 5 min to exchange information on the artists who produced the two paintings with fellow group members via an online chat program in order to choose the right answers. After the chat, they have to choose individually the artist responsible for each of such two additional paintings. Each correct answer is worth £1. The outcome of this task is only known at the end of the experiment together with the earnings from the selected decision.^13^

At the end of the experiment, after having completed the questionnaire, subjects in the painting and chat treatments received the answer key with the names of the artists who produced all the paintings.

Participants in the control group and in the anchoring treatment are not assigned to groups. A random matching is carried out simply for the purposes of analysis. The presence of a group to which they have been assigned is never communicated to subjects.

The first part of each session is the same for all groups: participants in all treatments and the control group play the BRET individually once. The treatment protocol is introduced in Part II of the experimental session and is also present in Part III. Thus, in Parts II and III, participants in the control group perform the BRET under the same conditions as in Part I. Instead, participants in the random, chat and painting treatments perform the BRET after receiving information about the number of boxes that each member of the group decided to collect on the previous occasion that the BRET was performed. Instructions make it clear to subjects that their payoffs depend solely on their own choices, not on the choices of other subjects. The information on choices made previously by group members is displayed both on the waiting screen before the main BRET screen and in the top right corner of the main BRET screen (above the fields where the participants have to write the number of boxes they would like to collect). The members assigned to a group in Part II are still in the same group in Part III.^14^

The anchoring treatment is similar to the control group because, in Parts II and III, subjects perform the BRET under the same conditions as in Part I. However, as in the other treatments, subjects see the previous choice of a—randomly assigned—peer group. Since the goal of the anchoring treatment is to distinguish the simple hint received from numbers from the willingness to be similar to assigned peers, information on peers’ previous choices is given through a different task where numbers are shown without reference to peers and subjects are not told that they are part of a group. Thus, participants in the anchoring treatment are asked about their painting preferences before performing the BRET. They are told that one hundred paintings by different artists have been selected (and are numbered from 1 to 100) and that they are going to see either two or three of these paintings, selected at random.^15^ They have to choose their favourite painting and will be informed about their preferred artist. The numbers of the selected paintings (that correspond to the previous BRET choices of a randomly assigned peer group) are displayed on both the waiting screen before the painting preferences elicitation and the feedback screen, which informs subjects of their favourite artist, coming immediately before the new BRET screen.

## Empirical analysis

### Summary statistics

Table 2 reports descriptive statistics for our indicator of risk behaviour and the dependent variable used to study peer effects, across treatment and control groups, before and after the implementation of the treatment protocol. It also reports two-sample t-tests for the equality of variable means between each treatment and the control group and an F-test for the equality of variable means across all groups. In this section and in the main analysis, we focus on just Parts I and II of each experimental session in order to avoid the well known reflection problem (Manski 1993) in estimating non-biased peer effects.^16^ We analyse data from Part III in Sect. 4.4.

**Table 2:** Descriptive statistics and treatment comparisons

	Control	Anchoring		Random		Painting		Chat		All
	Mean (SD)	Mean (SD)	t stat (p value)	Mean (SD)	t stat (p value)	Mean (SD)	t stat (p value)	Mean (SD)	t stat (p value)	F stat (p value)
Choice										
Part I	40.784 (18.590)	43.167 (22.231)	−0.600 (0.552)	38.396 (20.007)	0.614 (0.540)	43.611 (17.243)	−0.807 (0.422)	41.604 (16.567)	−0.232 (0.817)	0.60 (0.661)
Part II	46.275 (19.284)	41.185 (19.022)	1.361 (0.177)	42.542 (17.881)	0.999 (0.320)	46.167 (13.566)	0.033 (0.974)	42.542 (13.286)	1.127 (0.263)	1.00 (0.407)
Obs.	51	54		48		54		48		
GroupSD										
Part I	16.670 (5.800)	18.117 (10.222)	−0.509 (0.615)	18.716 (7.889)	−0.827 (0.415)	15.286 (5.791)	0.692 (0.494)	14.272 (7.091)	1.037 (0.308)	0.97 (0.429)
Part II	17.596 (7.040)	16.853 (8.136)	0.284 (0.778)	13.459 (7.278)	1.623 (0.115)	9.065 (6.033)	3.764 (0.001)	9.784 (8.035)	2.901 (0.007)	4.74 (0.002)
Obs.	17	18		16		18		16		

The second column in each treatment reports t statistic and p value of two-sample t tests for the equality of means between the corresponding treatment and the control group. The last column reports the F-stat and the p value of a test for the equality of variable means across all groups

The variable *Choice* represents the number of boxes that each student decides to collect and, therefore, his/her risk behaviour.

In Part I, where all subjects perform the task without being assigned to groups, the average number of collected boxes in the whole sample is 41.6. The majority of subjects (64.3 %) display risk averse behaviour (i.e. choose a number of boxes below 50); 12.6 % of the sample is risk neutral and the remaining 23.1 % choose to collect more than 50 boxes, thus displaying risk seeking behaviour.^17^ When looking at the average choice for the treatment and control groups separately, we see that the random treatment has the lowest average number of collected boxes (38) and that the anchoring and painting treatment have the highest average choice (about 43) while the control group and the chat treatment lie somewhere in the middle. Importantly, there are no significant differences across treatment and control groups in terms of subjects’ risk behaviour in Part I.^18^

In line with the findings of Crosetto and Filippin (2013), when subjects perform the task for a second time in Part II, after being grouped and having had information on the choices made in Part I by fellow group members, the average number of collected boxes overall (43.8) is significantly higher than in Part I^19^ (Wilcoxon signed-rank test p value = 0.0107) and subjects’ behaviour is slightly less risk averse: about 61 % is risk averse in Part II, 14.5 % is risk neutral and 24.5 % is risk seeking. There are no significant treatment differences in the average choice in Part II. A deeper analysis of the subjects’ risk behaviour is presented in Sect. 4.5.

To check the reliability of our variable *Choice* as a measure of individual’s risk behaviour, we compute its correlation with self-reported indicators of risk attitudes derived from answers to some questions from the final questionnaire: the general risk question used in the German Socio-Economic Panel (SOEP),^20^ that is “On a 0-10 scale, how do you see yourself: are you generally a person who is fully prepared to take risks or do you try to avoid taking risks?”, and similar domain-specific risk questions. We create risk indicators for both the general risk question and each of the domain-specific questions. These variables take values from 0 to 10 and increase with the propensity to take risk. We find that our measure *Choice* is positively and significantly (corr = 0.2714, p value = 0.000) correlated in Part I with the indicator of general risk attitudes.^21^ Moreover, we find evidence of a positive and significant correlation between our variable and the domain-specific risk indicators for all the domains: driving or cycling, financial matters, leisure and sport, occupation, studies, health, faith in other people (correlation and significance vary depending on the domain. The highest level of correlation is for occupation—corr = 0.2018, *p* value = 0.001—and the lowest is for leisure and sport—corr = 0.109, *p* value = 0.082).

Our main variable of interest is the standard deviation of the number of boxes chosen within a group (“*GroupSD*”). The higher *GroupSD* is, the higher heterogeneity within the group is. Since this variable is computed at the group level, all the analysis investigating the emergence of peer effects is carried out by using one observation for each group.

There are 85 groups in our dataset: 18 in the anchoring and painting treatments, 17 in the control group and 16 in both the random and the chat treatments.

In Part I, the standard deviation of subjects’ choices within the group they belong to is 16.67 on average for the control group, slightly higher for the anchoring and random treatment and slightly lower for both the painting and the chat treatment. However, it is never significantly different across treatment and control groups. In Part II, the average value of the variable *GroupSD* is substantially unchanged with respect to Part I for the control group, while a remarkable reduction of varying size can be observed for the four treatment groups. This is suggestive of the emergence of peer effects and of a possible role of group identity, which will be analysed in depth in the following section, where we report our main results. The F-test for the equality of variable means across all groups shows statistically significant differences between groups and two-sample tests for the equality of variable means between each treatment and the control group show a significant difference for the painting and chat treatments.

### Group identity and peer effects

In this section, we investigate the existence and the magnitude of peer effects on risk behaviour in order to answer our main research question: does the level of group identity affect the intensity of peer effects? That is, are people more likely to change their behaviour in order to conform with the behaviour of their peers when they feel a stronger sense of membership to the assigned social group?^22^

We compare behaviour in each of the three group identity treatments with behaviour in the control group and the anchoring treatment with the aim of verifying two hypotheses. Our first hypothesis concerns the existence of peer effects: if peer effects on risk behaviour exist, the behaviour of treated groups will differ from the behaviour of the control group and the anchoring treatment. Our second hypothesis concerns the role of group identity: if group identity affects the intensity of peer effects, the magnitude of the effect will increase as the level of group identity increases (i.e. it will be lower for the random treatment, higher for the painting treatment and even higher for the chat treatment).

We estimate the following linear regression model:

1 $$ Y_{g} = \alpha + \gamma A_{g} + \beta T_{g} + \varepsilon_{g} $$

where *Y*_*g*_ is the standard deviation of the choices of group *g* in Part II, when the treatment protocol is introduced; ${\text{A}}_{g}$ is the dummy for the anchoring treatment; *T*_*g*_ is a vector of dummies for the random, chat and painting treatments and *ε*_*g*_ is an error term.

The prediction is that, in the absence of peer effects, there should be no reason to expect the coefficients of the vector ${\text{T}}_{g}$ to be significantly different from zero and from $\gamma$. Moreover, if group identity plays no role in the intensity of peer effects, there should be no statistically significant difference between the different group identity treatments in the magnitude of the effects.

In column (1) of Table 3, OLS estimates of the above model are reported.^23^ Results show evidence of peer effects on risk behaviour. The standard deviation in the choices of groups of individuals in the random treatment is, on average, 4.1 boxes lower than the standard deviation in the choices of “fictitious” groups in the control, although the effect is not statistically significant (*p* value = 0.108). Participants in the painting treatment have a group standard deviation which is, on average, 8.5 boxes lower than the group standard deviation of subjects in the control group, with an effect that is statistically significant at the 1 per cent level. The reduction in the standard deviation of group choices with respect to the control is very similar (7.8) for the chat treatment and the effect is again significant at the 1 per cent level. The coefficient of the anchoring treatment is negative, but very small and not statistically significant. The reduction in the standard deviation experienced by both the painting and the chat treatment is significantly larger than the anchoring treatment (*F*-stat = 10.21 and 6.22, *P* value = 0.002 and 0.015, respectively). Thus, what we observe in our data is a wish to be similar to the assigned peers and not simple anchoring to given numbers.

**Table 3:** Group identity and peer effects

	Group standard deviation		
	Part II	Part I	Difference
	(1)	(2)	(3)
Anchoring	−0.7433	1.4477	−2.1910
	(2.6215)	(2.8487)	(3.7205)
Random	−4.1368	2.0464	−6.1832**
	(2.5459)	(2.4715)	(2.8217)
Painting	−8.5312***	−1.3836	−7.1476**
	(2.2684)	(2.0010)	(3.0361)
Chat	−7.8124***	−2.3978	−5.4146
	(2.6899)	(2.3088)	(3.3113)
Observations	85	85	170
Adj R^2^	0.151	−0.001	0.111

OLS estimates

The symbols ***, **, * indicate that the coefficients are statistically significant at the 1, 5 and 10 % level, respectively

Moreover, our results show that group identity strengthens peer effects: an *F*-test for the equality of the reduction in the group standard deviation experienced by the random and the painting treatments shows a statistically significant positive difference: *F*-stat = 3.48, *P* value = 0.066. A similar test comparing the painting and chat treatments shows no statistically significant difference (*F*-stat = 0.08, *P* value = 0.775). A possible explanation for this is that the perceived behaviour of other group members in this task may either enhance or mitigate (or leave unaffected) the feeling of belonging to the group. Thus, the lack of an additional significant effect may be due to the average effect being estimated. A deeper analysis of this issue is presented in the following section.

In column (2), we run the same specification as column (1) by using data from Part I, when all individuals perform the task under the same conditions and no treatment protocol is introduced. As expected, the coefficients of the treatment dummies are always not significantly different from zero and of a similar size.

Whereas Chen and Li (2009) found that pure categorization itself is sufficient to create group effects, because a random assignment is as effective as a group assignment based on participant painting preferences in shaping social preferences, our results show that, the typology of categorization matters when looking at an individual’s risk behaviour and individuals are more affected by their peers when the group assignment is based on painting preferences rather than being random.

Much as in Chen and Li (2009), the group task does not increase peer effects. In the following section, we explore possible explanations for why we do not see a treatment effect for the chat treatment and show some circumstances under which the group task may contribute to strengthening peer effects.

Given the small sample size, we check the robustness of our results in the final column of Table 3 by estimating a difference-in-differences model, in order to extrapolate the effect due to the treatment protocol alone. Coefficients in the table represent the difference between *ex post* (Part II) differences in the group standard deviation between the control group and each of our four treatments and the corresponding *ex ante* (Part I) differences.^24^ The effects are less precisely estimated, but results are consistent with the findings in columns (1) and (2): even when we exclude the *ex ante* (not statistically significant) differences between the control group and our treatments, we find a negative effect of the treatment protocol on the standard deviation of group members’ choices (*p* values of the coefficients are 0.031, 0.021 and 0.106 for the random, painting and chat treatments, respectively). Again, peer effects are slightly larger in the painting than in the random treatment (although the difference is no longer statistically significant), while the chat treatment does not produce a significant increase in the effect.

It is worth noting that the standard deviation of group choices in the control group does not change significantly across the two parts.^25^ Given that participants in the control group are never exposed to feedback on group choices and are only randomly assigned to groups for the purposes of analysis, this result makes us confident that what we are observing is not spurious convergence towards a particular value in the second repetition of the task, but is convergence generated by the knowledge of peers’ decisions and the desire to be similar to them.

### Feelings of attachment and treatment effects

In this section, we explore a possible explanation for why we do not see an additional treatment effect for the chat treatment. We speculate that there is a potential issue with regard the inducing of a higher level of group identity by having subjects perform a group task before the BRET given that the behaviour of individuals in the group task (for example absence of collaboration) may weaken the sense of belonging to the group instead of strengthening it. Besides spending more time with their assigned group, individuals receive new information about their peers during the group task that can either strengthen or weaken their perceived similarity to fellow group members. Therefore, depending on personal experience during the group task, perceived group identity might be either enhanced or mitigated and the lack of an additional significant effect may be due to the average effect being estimated.

To investigate this issue, we restrict our attention to the chat treatment and to a set of group characteristics that capture information about the quality of personal experience during the group task which we speculate might relate to perceived group identity. On the basis of the considered characteristics, we split groups in the chat treatment into those where group identity may have been enhanced by the group task (*ChatX*) and those where the realization of the considered characteristics could have weakened group identity (*Chat*), thus estimating the following model: $Y_{g} = \alpha + \beta \;Chat_{g} + \theta \;ChatX_{g} + \varepsilon_{g}$. OLS estimates are shown in Table 4.

**Table 4:** Feelings of attachment and treatment effects

	Group standard deviation			
	(1)	(2)	(3)	(4)
Chat	−7.1606**	−6.2873*	−4.4881	−4.1849
	(2.9233)	(3.1233)	(3.6472)	(4.1434)
Chat helpful group	−12.3752***			
	(1.8850)			
Chat attached to group		−12.3878***		
		(2.3486)		
Chat ≤ median minutes for agreement			−9.8128***	
			(3.1668)	
Chat ≤ median number of messages for agreement				−9.4233***
				(2.9313)
Observations	33	33	32	32
Adj R^2^	0.184	0.213	0.189	0.186

OLS estimates

The symbols ***, **, * indicate that the coefficients are statistically significant at the 1, 5 and 10 % level, respectively

Firstly, we take into consideration a question from the final questionnaire which asks subjects to rate, on a scale from 0 to 10, how much they thought communicating with their group members helped solve the two extra painting questions in the group task.^26^ We use the answers to this question to estimate, in column (1), peer effects on risk behaviour in the chat treatment separately for those groups whose participants felt that the level of help received from fellow group members during the online chat was higher than the average level, *Chat Helpful Group,* 27 and for the remaining groups (coefficient on the variable *Chat*). Groups whose participants considered their group to be more helpful than the average have a group standard deviation which is, on average, 12.4 boxes lower than the group standard deviation in the control group, an effect which is statistically significant at the 1 % level, about 5.2 boxes larger than the effect for the remaining groups in the chat treatment and 3.8 boxes significantly larger than the effect of the painting treatment (*F*-stat = 5.83, *p* value = 0.0181). Since the group task is likely to have enhanced the level of group identity for these groups in the chat treatment, this result confirms our idea that personal experience in the group task and additional information on group members gathered when interacting more may have both positive or negative effects on perceived group identity that may compensate for one another when an average effect is estimated.

Next, we use another question from the final questionnaire which asks subjects to rate, on a scale from 0 to 10, how closely attached they felt to their own group throughout the experiment. This is used to identify groups where at least two members rated their attachment to their assigned peers at a higher level than the average (3.3), *Chat Attached to Group*.^28^ Estimates in column (2) show that the effect for these groups is −12.4 boxes, statistically significant at the 1 % level. This effect is 6.1 boxes larger than the effect for the remaining groups in the chat treatment and about 3.9 boxes significantly larger than the effect of the painting treatment (*F*-stat = 3.34, *p* value = 0.071).

The time subjects spend chatting and the number of messages they exchange in order to reach an agreement on the answers in the group task are two additional indicators of the extent to which the group task actually contributes to the strengthening of group identity. Indeed, an increase in the time (and number of messages) needed to find a common answer to the questions signals a higher diversity of opinions among group members and greater difficulty in converging towards a common view. At one extreme, members of groups that are not able to find an agreement may even find that the group task weakens any sense of group attachment they might have. In columns (3) and (4), we estimate peer effects on risk behaviour separately for groups that, in order to reach an agreement, needed to chat for a number of minutes that is lower (*Chat* ≤ *Median Minutes for Agreement*) or higher (*Chat*) than the median (1.3) and needed to exchange a number of messages that is lower (*Chat* ≤ *Median Number Of Messages for Agreement*) or higher (*Chat*) than the median (9), respectively.^29^ Both estimates show that the magnitude of peer effects is higher when the time or the number of messages needed to reach an agreement on the possible answers in the group task is lower than the median, possibly because individuals feel more similar to their assigned peers.

Overall our estimates suggest that the weaker peer effects found for the chat treatment are due to the fact that the group task does not always enhance the level of group identity. When the level of group identity actually increases across treatments, so does the magnitude of peer effects on risk behaviour. It is worth noting that being able to investigate further how different individual experiences in the group task influence perceived group identity and, in turn, its impact on peer effects comes at the price of introducing some endogeneity into our model. Indeed, in such estimates we cannot exclude the existence of an omitted variable which correlates with both our outcome variable and the perceived (helpfulness, attachment) or actual (time and messages to reach an agreement) experience in the group task.

### Peers’ influence over time

In this section, we extend our analysis by also looking at data from Part III of the experimental session, where individuals are in the same group as they were in Part II and perform the BRET another 10 times. On each of these occasions, they have information on the number of boxes that each group member decided to collect in the previous repetition of the task.

In Table 5, we estimate the same specification as in column (1) of Table 3 30 by considering both Parts II and III, that is all the repetitions of the task in which participants have information on group members’ previous choices (column 1), and data from Part III only, to check whether peer effects are short lived or longer lasting (column 2). There are no anchoring effects in either regression and the coefficients of our group identity treatment dummies are significantly different from zero.^31^ This confirms our result that individuals are affected by their peers when taking decisions in risky settings and shows that peer influence does not vanish after the first interaction.^32^

**Table 5:** Peer effects in further repetitions of the task

	Group standard deviation			
	Part II + Part III	Part III	Part II + Part III	Part III
	(1)	(2)	(3)	(4)
Anchoring	−1.6957	−1.7910	−1.6957	−1.7910
	(1.8945)	(1.9242)	(1.8955)	(1.9253)
Random	−5.9115***	−6.0889***	−5.9115***	−6.0889***
	(1.9670)	(2.0176)	(1.9681)	(2.0188)
Painting	−7.2910***	−7.1669***	−7.2910***	−7.1669***
	(1.7612)	(1.8145)	(1.7622)	(1.8155)
Chat	−6.8399***	−6.7427***	−6.8399***	−6.7427***
	(1.9748)	(1.9891)	(1.9758)	(1.9902)
Repetition number			−0.1229	−0.2471**
			(0.0855)	(0.0975)
Observations	935	850	935	850
Adj R^2^	0.114	0.109	0.115	0.115

OLS estimates

The symbols ***, **, * indicate that the coefficients are statistically significant at the 1, 5 and 10 % level, respectively

Results remain the same even when we control for the number of the repetition within the sequence of 11 (Part II + Part III) or 10 (Part III) repetitions of the task (columns 3 and 4). In particular, for Part III, each repetition of the task further reduces the group standard deviation by 0.2 boxes on average with an effect that is significant at the 5 % level.^33^

### Relative risk behaviour and change in individuals’ decisions

In this section, we want to investigate whether, when making their choices, individuals are influenced by their riskiness rank within the three-person group they belong to—something that can be easily figured out from the feedback on group members’ previous decisions—and whether different rank positions are associated with different systematic behaviours. As in our main analysis, we only use data from Parts I and II in this section.

On the basis of the choice made in Part I, a student can find him/herself matched with two peers that are either both riskier or both less risky than him/her or with peers that are mixed in terms of riskiness (one riskier and one less risky than him/her).

**Fig. 3:**
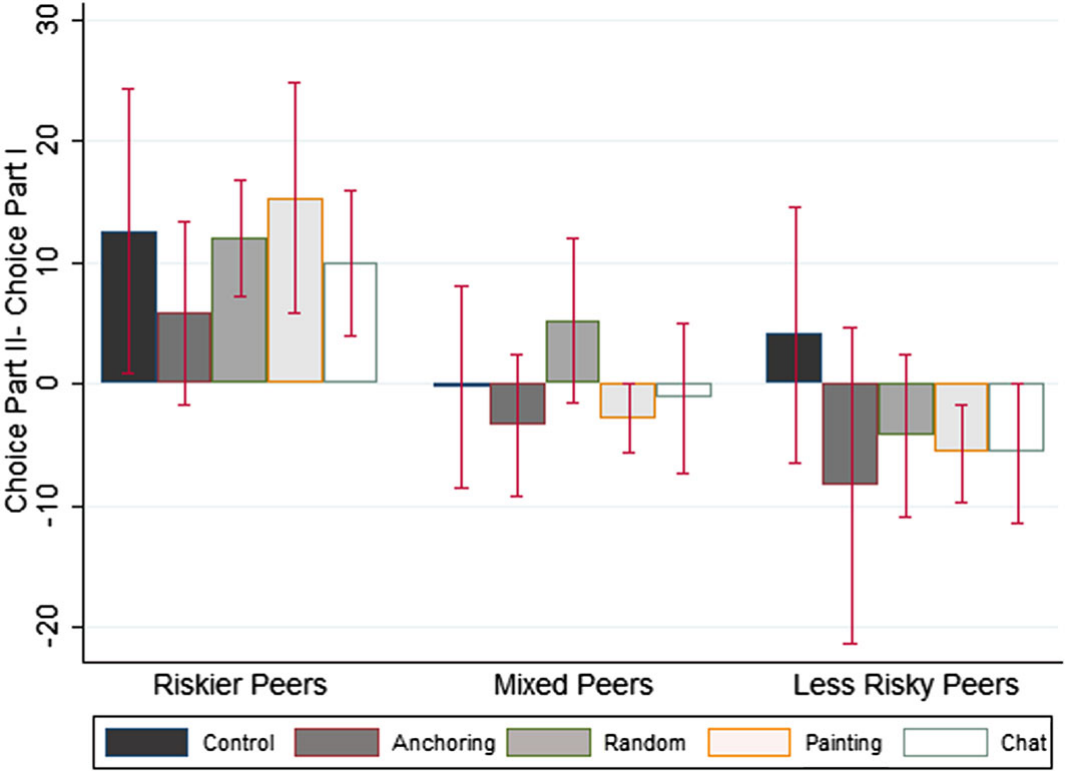
Rank position in Part I and change in the number of collected boxes

Figure 3 shows, separately for treatment and control groups, how the number of boxes collected in Part II differs from the decision in Part I for each of the three typologies of peers that a student may face. On the one hand, when a student in the random, chat or painting treatment is assigned to a group whose members are both riskier than him/her, that is s/he is the bottom ranked in terms of the number of boxes collected when the BRET task is performed for the first time, s/he tends to significantly increase the number of boxes collected in Part II. On the other hand, being the top ranked member of the group, so having two less risky peers, is related to a reduction in the number of boxes collected in the second repetition of the task: on average the difference between the second and first choice is negative and is statistically significant for the painting treatment. Finally, the change in choice for subjects with mixed peers is always not significantly different from zero. For completeness, we report data from the control group and anchoring treatment as well. For these subjects, the effects are very imprecisely estimated and not significantly different from zero.

In Table 6, we use individual level data in Part II of the experiment to run OLS estimates of a linear regression model with the aim of investigating whether different rank positions within the group, determined on the basis of the choices made in Part I, are associated with different systematic behaviours.

**Table 6:** Rank position in Part I and number of collected boxes

	Choice part II–choice part I				Choice part II	
	Whole sample	Control	Anchoring	Group identity treatments	Group identity treatments	Subsample of group identity treatments
	(1)	(2)	(3)	(4)	(5)	(6)
Less risky peers	−3.6556*	4.3529	−5.0667	−5.5336***	−1.0027	2.1146
	(1.9401)	(4.6598)	(5.6511)	(1.9787)	(1.9885)	(2.8963)
Riskier peers	11.6264***	12.8235**	9.1754**	12.2818***	6.9330***	5.4741**
	(1.9212)	(5.5615)	(3.6232)	(2.3836)	(2.1448)	(2.4573)
Choice part I					0.6685***	0.5228***
					(0.0722)	(0.1099)
Observations	255	51	54	150	150	50
Adjusted R^2^	0.143	0.031	0.053	0.278	0.439	0.149

The symbols ***, **, * indicate that the coefficients are statistically significant at the 1, 5 and 10 % level, respectively

In columns (1) to (4), we use the change in choice between the two parts as the dependent variable and dummies for the typology of peers that a student may face (mixed peers is the reference category) as control variables. Estimates confirm what has been shown by the graph: on the one hand, individuals whose peers are both less risky decrease their choice in Part II by about 3.7 boxes on average compared with individuals with mixed peers; on the other hand, individuals whose peers are both riskier tend to increase their choice by about 11.6 boxes compared with people who have mixed peers. When looking at relative risk behaviour for the control group, the anchoring treatment and the group identity treatments separately, we find that individuals assigned to less risky peers in the random, painting and chat treatment reduce their choice significantly by about 5.5 boxes and individuals assigned to riskier peers increase their choice significantly by about 12.3 boxes. Similar effects are observed in the anchoring treatment although the effect for subjects who have less risky peers is very imprecisely estimated. As regards the control group, the coefficients are positive regardless of the typology of peers that participants engage with (although the effect is not significant for subjects who have less risky peers). This may possibly reflect the general trend of reduced risk aversion in the second repetition of the task with a larger reduction for very risk averse individuals.^34^

Using the change in choice between the two parts as the dependent variable means assuming persistence in risk behaviour, in other words forcing the coefficient on the choice that is made the first time the task is performed to be 1. In column (5), we relax this assumption and run a regression which has the number of boxes collected in Part II as the dependent variable and introduces the number of boxes collected in Part I among the regressors. This specification, estimated on the subsample of individuals in the group identity treatments, allows us to separate peer effects on risk behaviour from mean regression effects. The coefficient on *Choice Part I* shows that part of the observed effect is due to regression to the mean: subjects who collected one additional box in Part I will collect 0.7 additional boxes on average in Part II. However, even when controlling for regression to the mean, peers’ risk behaviour plays a role in shaping an individual’s decision in Part II if the student has two riskier peers: his/her choice in Part II increases by about 7 boxes with an effect that is statistically significant at the 1 % level. Subjects with two less risky peers decrease their choice on average, but the effect is not significantly different from zero.^35^

In column (6) we check the robustness of our results by studying the effect of ending up in different riskiness rank positions within the group in the subsample of participants belonging to the group identity treatments whose initial risk attitude is at a level for which we have at least one subject for each riskiness rank. We find that participants who start from the same risk attitude and end up having riskier peers significantly increase their choice in Part II by 5.5 boxes. A significant role of peers’ risk behaviour for subjects with riskier peers is found also when considering the change in choice between the two parts as the dependent variable.

All in all our estimates suggest that subjects tend to adjust their choice on the basis of their relative position: individuals whose peers are both riskier/less risky tend to increase/decrease their choice in Part II when compared with individuals with mixed peers. When ruling out the component of the effect caused by regression to the mean, peers’ risk behaviour continues to play a significant role for bottom ranked individuals while it is very close to zero for top ranked individuals.

## Concluding remarks

In this paper, we study whether and to what extent group identity affects the magnitude of peer effects on an individual’s risk behaviour. We believe our paper is the first to provide evidence of how different perceptions of membership to an assigned social group can affect the tendency of individuals to change their risk behaviour in order to match the prevalent behaviour of their peers.

We run a laboratory experiment where an individual’s risk behaviour is measured by using the Bomb Risk Elicitation Task. Peer effects are introduced by giving subjects information on their peers’ previous decisions and different levels of group identity are induced by combining two assignment methods (one random and one based on painting preferences) and a collective problem solving task that uses an online chat program to enhance the feeling of belonging to the assigned group. The presence of anchoring effects is controlled by running a treatment where individuals are shown numbers without any reference to a peer group and peers’ choices.

We find that subjects are affected by their peers’ choices and they change their decisions in order to assimilate their behaviour to that of their peers when they have information on the choices made by the fellow members of their group. This change in behaviour is not driven by anchoring, but represents pure peer effects. Moreover, the typology of categorization matters and individuals are significantly more affected by their peers when the group assignment, instead of being random, is based on painting preferences, that is group identity is intensified because group members are more likely to feel similar to each other due to their sharing at least one characteristic with their peers. We do not observe a significant increase in the magnitude of peer effects when the less impersonal preference-based matching procedure is combined with a group task. We speculate that this may be due to the fact that the group task does not always contribute to the enhancing of group identity and find that the magnitude of peer effects increases when the group task strengthens the feeling of group belonging because group members feel attached to each other, help each other during the online chat and/or the group reaches an agreement on the possible answers to the group task very quickly.

Peer effects are persistent over time while the effect of personal experience during the group task on perceived group identity, and in turn on peer effects, is weakened with further repetitions of the risk elicitation task. Moreover, we find that individuals are influenced by their riskiness rank within the three-person group they belong to, which they can easily work out from the feedback on group members’ previous decisions. Individuals whose peers are less risky tend to make less risky choices, while individuals matched with riskier peers tend to take on more risk. This latter effect remains significant even when ruling out the component of the effect due to regression to the mean.

Studying the influence peers have on an individual’s risk behaviour may provide an important contribution to the optimal design of policy interventions aimed, for example, at remediating deficits in educational achievement and improving the labour market prospects of the young unemployed. We show that peers influence an individual’s risk behaviour in an artificial social environment, i.e. a laboratory setting, and that a less impersonal categorization procedure and an increased—positive—interaction with fellow group members strengthen peer effects. Our results may be indicative of the effect peer group behaviour may have on an individual’s risk behaviour in the real world too. This would imply that targeting the risk behaviour of a few individuals may have an effect on outcomes which have been found strongly influenced by risk, not only for the target pool of subjects, but also for their peers. Moreover, given that the magnitude of peer effects increases as the closeness to the group increases, choosing the target pool wisely may amplify the benefits of the policy intervention.

Our result that more interaction among peers is, in itself, not sufficient to enhance the salience of group identity makes the investigation of the dynamics of peer interaction and of the influence of new information on group members interesting. Moreover, our experiment focuses on risk behaviour by referring to monetary decisions in the gain domain. Risk matters across a whole range of other domains, too (i.e. monetary-loss domain, health, leisure and sport, occupation, etc.). Hence, studying the role of group identity and peer effects on a person’s decision making in other risk domains would appear to be a fruitful avenue for future research.

### Electronic supplementary material

Below is the link to the electronic supplementary material.

Supplementary material 1 (DOC 92 kb)

## Appendix 1: Randomization checks

In the first five columns in Table 7, the means for a number of individual characteristics are reported in treatment and control groups separately. In the last two columns, we report the *F*-stat and the *p* value of a test for the equality of variable means across all groups.

**Table 7:** Participants’ characteristics across treatment groups

	Means					F-stat	p value
	Control	Anchoring	Random	Painting	Chat		
Choice	40.784	43.167	38.396	43.611	41.604	0.603	0.661
Female	0.647	0.648	0.646	0.630	0.75	0.511	0.728
Age	22.647	22.241	22.688	22.685	22	0.184	0.946
Asian	0.333	0.333	0.417	0.296	0.271	0.663	0.618
European	0.588	0.611	0.5	0.593	0.604	0.405	0.805
Economics	0.196	0.278	0.208	0.241	0.167	0.542	0.705
HSS	0.627	0.556	0.563	0.556	0.625	0.290	0.884
MVM	0.078	0.000	0.042	0.093	0.104	1.611	0.172
Distinction	0.235	0.315	0.271	0.407	0.271	1.099	0.357
Good	0.627	0.519	0.479	0.444	0.521	0.973	0.423
Mother uni degree	0.529	0.500	0.646	0.519	0.625	0.900	0.465
Father uni degree	0.667	0.556	0.75	0.648	0.646	1.072	0.371
Brothers	0.824	0.741	0.625	0.852	0.667	0.604	0.660
Sisters	0.725	0.759	0.729	0.611	0.563	0.585	0.674
Self-reported risk	5.333	5.481	5.125	5.537	5.521	0.307	0.873
Smoker	0.137	0.111	0.063	0.093	0.125	0.441	0.779
Drinker	0.922	0.889	0.875	0.870	0.938	0.470	0.758
Study alone	0.863	0.926	0.896	0.926	0.813	1.148	0.334
Observations	51	54	48	54	48		

In the last two columns, we report the *F*-stat and *p* value of a test for the equality of variable means across all groups

*HSS* is the humanities and social sciences degree, *MVM* is the medicine and veterinary medicine degree

## Appendix 2: Peer influence in Part III: effect of repetition number

In Table 8, we regress our dependent variable *GroupSD*, separately for each treatment, on a categorical variable for the repetition number of the task in Part III of the experiment. We find that, on average, the group standard deviation of subjects in the random and painting treatments diminishes by about 0.3 and 0.4, respectively, for each repetition of the task in Part III of the experiment, with an effect that is significant at the 10 % level. No significant effect emerges for subjects in the anchoring and chat treatments or in the control group.

**Table 8:** Effect of repetition number in Part III

	Group standard deviation				
	Control	Anchoring	Random	Painting	Chat
Repetition number	−0.2461	−0.3613	−0.3469*	−0.4037*	0.1564
	(0.2364)	(0.2535)	(0.1963)	(0.1948)	(0.1905)
Observations	170	180	160	180	160
Adj R^2^	0.001	0.007	0.011	0.020	−0.003

OLS estimates

The symbols ***, **, * indicate that the coefficients are statistically significant at the 1, 5 and 10 % level, respectively

## Footnotes

1 The psychology and economics literature shows that time pressure is detrimental for decision quality (Diederich 1997; Busemeyer and Diederich 2002; Diederich and Busemeyer 2003) and for performance (De Paola and Gioia 2016).

2 Research using the priming method has shown that making different natural social identities salient through priming can affect outcomes, such as test performance or walking speed (Steele and Aronson 1995; Shih et al. 1999; Spencer et al. 1999; Fershtman and Gneezy 2001; Yopyk and Prentice 2005; Bernhard et al. 2006; Götte et al. 2006; Bargh 2006; McLeish and Oxoby 2011). However, the priming method may lack some of the desired control over the experimental situation and conclusions drawn from using this method may be hampered by confounding factors such as experimenter demand effects and spuriously induced behaviour changes (Zizzo 2012).

3 Social interaction may influence individual behaviour for many reasons. We do not aim to identify the specific mechanism at work in our setting however the most likely one is conformism, the act of changing one’s behaviour to match the responses of others (Cialdini and Goldstein 2004; Asch 1955). If conformism is at work, the extent to which individuals like to behave similarly to their peers may be stronger, the deeper the sense of belonging to the group is. The scope for social learning is reduced to a minimum by our design and if it plays any role, given that the available information on peer behaviour is the same, its effect is plausibly similar across group identity treatments. Social utility in the form of relative payoff concerns and, thus, potential feelings of envy or guilt (Fehr and Schmidt 1999) is unlikely in our setting because there is no direct spillover across individuals’ payoffs and, even if they make the same choice, there is a very low probability of their earning the same payoff. Participants do not directly interact before performing the task, so there are no knowledge spillovers that involve direct sharing of information. Moreover, individuals and groups are anonymous, therefore there is no way of expressing approval or disapproval which might give rise to a social norm and there is no social pressure arising from the fear of being alone, marginalized by the peer group and/or considered "different".

4 Moreover, unlike other risk elicitation methods, such as the Balloon Analogue Risk Task (Lejuez et al. 2002), there is no truncation of data in the BRET.

5 By comparing different treatments, Chen and Li (2009) find that, on one hand, random assignment is as effective as group assignment based on participant painting preferences in inducing group identity; on the other hand, the group task using the online chat significantly increases the self-reported attachment to the group and might have a moderate effect on behaviour. The authors also introduce other-other allocation tasks and find that they have no significant effect. Based on these results and on the authors' concern that the group effect induced by categorization may deteriorate over time, we decide not to introduce the other-other allocation tasks and to reduce the length of the chat in the group task from 10 to 5 min.

6 We asked participants to write the same number twice and to confirm their choice in order to avoid measurement errors due to an incorrect number being input.

7 We decided in favour of a static version of the BRET to avoid individual levels of impatience affecting participants’ decisions and, thus, our indicator of risk behaviour (in the dynamic version of the BRET, one cell is automatically deleted from the screen each second, to represent a collected box, and subjects have to wait while the deletion process goes on until their chosen number of boxes have been collected. Even when the time interval between the deletion of the cells is reduced, this mechanism always entails an element of patience whereby a high level of risk aversion might reflect lower patience and vice versa). Nevertheless, given that Crosetto and Filippin (2013) point out higher comprehension problems in the static version, in order to be sure that a participants’ decision was not driven by confusion or imperfect comprehension of outcomes and probabilities, we also introduced a dynamic visual representation of the collection process when explaining the rules of the task and a visual representation of the boxes collected and uncollected after each decision.

8 We decided to determine the position of the bomb individually because recent evidence on the BRET (Crosetto and Filippin 2015b) shows that there seem to be peer effects when the random draw is carried out once for the whole lab rather than individually.

9 According to Tversky and Kahneman (1974) the anchoring effect is the disproportionate influence on decision makers to make judgments that are biased toward an initially presented value. The anchoring effect has been studied in different domains, such as valuations and purchasing decisions (Wansink et al. 1998; Mussweiler et al. 2000; Ariely et al. 2003). See Furnham and Boo (2011) for a review.

10 The sessions of the Anchoring treatment were conducted in October 2015.

11 As in Chen and Li (2009), the five pairs of paintings are: 1A Gebirgsbildung, 1924, by Klee; 1B Subdued Glow, 1928, by Kandinsky; 2A Dreamy Improvisation, 1913, by Kandinsky; 2B Warning of the Ships, 1917, by Klee; 3A Dry-Cool Garden, 1921, by Klee; 3B Landscape with Red Splashes I, 1913, by Kandinsky; 4A Gentle Ascent, 1934, by Kandinsky; 4B A Hoffmannesque Tale, 1921, by Klee; 5A Development in Brown, 1933, by Kandinsky; 5B The Vase, 1938, by Klee.

12 As in Chen and Li (2009) the two additional paintings are: Monument in Fertile Country, 1929, by Klee and Start, 1928, by Kandinsky.

13 56.25 % of the participants provided correct answers to both paintings; 6.25 % provided one correct answer and 37.5 % provided zero correct answers.

14 Since one of the aims of inducing a growing sense of group identity is to extrapolate our insights into the real world, where people often self-select into groups, we could have added another treatment requiring participants to invite two other people (maybe of the same gender) to the session and let these three people constitute a peer group, as in Gardner and Steinberg (2005). We decided not to introduce such a treatment because people in our setting do not know that they will be matched into groups until they are actually assigned to groups (Part II). People invited to come with friends may presume that part or all of the experiment will involve some interaction with their friends and this may condition their behaviour, even in Part I when they perform individually.

15 Instructions stated “either two or three paintings” because, if two of the previous choices were the same, subjects saw only two paintings. A case in which all three made the same choice did not occur.

16 The reflection problem arises when peers interact repeatedly because if an individual’s choice responds to his/her peers’ choices, then the peers’ subsequent choices will reflect the individual’s own previous choices.

17 See Crosetto and Filippin (2013) for details on how to formalize subjects’ decisions in the BRET.

18 We present our randomization checks in Appendix 1. Table 7 shows no systematic differences in any of the individual characteristics, including those that could be correlated with risk taking behaviour outside the lab, such as smoking. We also performed additional tests that confirm the absence of *ex ante* heterogeneity in risk behaviour across treatment and control groups (a two-sample Kolmogorov–Smirnov test for equality of distribution functions and an Epps-Singleton two-sample empirical characteristic function test). Similarly, the two-sample Wilcoxon rank-sum (Mann–Whitney) test confirms the absence of significant difference in the choice in Part I between any two groups (Results not reported and available upon request).

19 The same results hold true when we regress the number of collected boxes on the dummy for Part II.

20 The validity of using the self-reported general risk question as opposed to the results of incentivized lottery-based tasks to elicit risk attitudes has been explored by Dohmen et al. (2011), who show that self-reported answers can represent a valid low-cost substitute for incentivized lottery schemes.

21 The correlation coefficients are higher than those usually found in the literature (Deck et al. 2013; Charness and Viceisza 2015; Crosetto and Filippin 2015a). We investigate possible reasons. If we consider the average choice over the 12 BRET repetitions, the correlation of this variable to the indicator of general risk attitude is higher (corr = 0.3851, p value = 0.000). The two correlation coefficients are significantly different at a 5.14 % level. However, this result is driven by group identity treated subjects: the correlation coefficient increases from 0.2113 (p value 0.009) to 0.3696 (p value 0.000) when considering *Choice* in Part I and the average choice over the 12 BRET repetitions, respectively, with a difference that is significant at the 4.36 % level. The increase in the correlation coefficient value is not significant for the control group and the anchoring treatment. Thus, our data would suggest that the self-reported risk attitude measurement is affected by having been exposed to several BRET repetitions and possibly having converged to a risk attitude which is somewhat more in common with that of fellow group members. However, although correlation coefficients differ according to the variable considered (*Choice* in Part I vs average choice), especially for treated subjects, they do not differ across treatments: we fail to reject the null hypothesis of equal correlation matrices for treated, control and anchoring subjects, either when using *Choice* in Part I (p value 0.400) or when using the average choice over the 12 repetitions of the BRET (p value 0.104).

22 A simple model describing behaviour in our setting assumes that the individual chooses his/her optimal action by maximizing an additive utility function, such as $U_{i} = \pi \left( {x_{i} } \right) - \beta \left( {GI_{i} } \right)d\left( {x_{i} ,x_{j} } \right)$ where the first term is a payoff deriving from the choice of *x*_*i*_ and the second term is the loss in utility arising from making a different choice from *x*_*j*_, that of other group members, weighted by a function of the level of group identity, $\beta \left( {GI_{i} } \right)$.

23 In all estimates, we cluster observations at the group level. Moreover, results are also the same when checking for session random effects.

24 In terms of our difference-in-differences model, $Y_{gt} = \alpha + \gamma A_{g} + \beta T_{g} + \delta PartII_{t} + \varphi \left( {A_{g} *PartII_{t} } \right) + \theta \left( {T_{g} *PartII_{t} } \right) + \varepsilon_{gt}$, the coefficients in the table are the coefficients of the interaction terms between each treatment and the dummy for Part II (*φ* and the vector *θ*).

25 That is δ, the coefficient on dummy *Part II* in the difference-in-differences model in footnote 24, is not significantly different from zero.

26 On average, subjects rate the help received from their group at the level of 5.5.

27 On average 12.5 % of the groups in the chat treatment perceive their group to be more helpful than the average in the group task.

28 On average 25 % of the groups in the chat treatment have at least two members who expressed a level of attachment above the average. There are no groups where all the three members declared an above average level of attachment.

29 In our sample, only one group out of 16 did not reach an agreement on the name of the artists responsible for the two additional paintings shown in the group task. We read the log files of the chat process for the remaining 15 groups to compute two indicators of the difficulty in reaching an agreement (and, thus, weaker feelings of belonging to the group). On average, subjects need about 1.8 min and 9.6 messages to agree on an answer for the group task. Results also hold true when considering the mean instead of the median. In order to compute these indicators, we do not take into consideration time and messages that were used for purposes other than to agree on the task answers (i.e. greetings and comments). In total, subjects chatted for about 3.1 min and exchanged 13.6 messages on average.

30 We obtain similar results when we estimate the difference-in-differences model as in column (3) of Table 3.

31 The magnitude of the effect in the painting treatment is larger than in the random treatment, but the difference is not statistically significant.

32 When we replicate the specification in column (1) of Table 4, we find that the effect on perceived group identity, produced by personal experience in the group task and additional information on group members gathered during greater interacting, attenuates over time and is no longer statistically significant.

33 When, separately for each treatment, we regress our dependent variable *GroupSD* on a categorical variable for the repetition number of the task in Part III of the experiment, we find that, on average, the group standard deviation of subjects in the random and painting treatments is reduced by 0.3 and 0.4, respectively, for each repetition of the task in Part III of the experiment, an effect which is significant at the 10 % level. No significant effect emerges for subjects in the anchoring and chat treatments or in the control group. Results are reported in Appendix 2, Table 8.

34 Results hold true when we run the specification in column (1) adding the interaction terms between the risky behaviour of the group and a dummy for being in the control group: the interaction term is not statistically significant for subjects having riskier peers but it is positive and significant for subjects whose peers are less risky.

35 Our results remain qualitatively unchanged if, instead of considering a dummy for the individual’s rank within the group based on the level of riskiness of peers, we look at how far the top and bottom ranked subjects are from either the choice of the middle ranked student or the mean choice of their group.
