# Supplementary material for: Peer effects on risk behaviour: the importance of group identity
Source: Exp Econ. 2016 Mar 18;20(1):100–29. doi: 10.1007/s10683-016-9478-z (PMC5326808; doi:10.1007/s10683-016-9478-z)
Supplement: Supplementary file 1 — Supplementary material 1 (DOC 92 kb) [file 10683_2016_9478_MOESM1_ESM.doc]

**Experimental Economics**

**PEER EFFECTS ON RISK BEHAVIOUR: THE IMPORTANCE OF GROUP IDENTITY**

**Francesca Gioia*[[1]](#footnote-2)****

*School of Economics, University of Edinburgh*

**EXPERIMENTAL INSTRUCTIONS**

*Materials inside the square brackets are not displayed on the subject instructions.*

Welcome. Thank you very much for agreeing to participate in today’s experiment.

Please refrain from talking or trying to communicate with any other participants until the experiment is finished. Also, please remember to turn off your mobile phones. Participants intentionally violating the rules may be asked to leave the experiment and may not be paid.

The purpose of this experiment is the analysis of economic decision making.

The experiment lasts for maximum one hour and consists of three parts. Your decisions in one part have no implications for your decisions in the other parts. You will receive detailed instructions at the beginning of each part and, when needed, before each relevant step in the experiment. The experimenter will read the instructions aloud and you will see them on your screen. Please, press the button OK only when you have finished reading the instructions to go ahead with the experiment.

The experiment ends with a short questionnaire and, after having answered the questionnaire, you will see your earnings displayed on your screen.

If you have any questions or problems at any point in today’s experiment, please raise your hand and one of the experimenters will come to you and answer your questions in private at the end of the instructions.

**Payoff**

The experiment is funded by the School of Economics of the University of Edinburgh.

During the experiment your income is directly calculated in Pounds.

At the end of the experiment, one of your decisions will be selected at random by the computer for payment. We will calculate your earnings based on the selected decision. These earnings will be shown on your screen together with the selected decision.

Your earnings will be paid out in cash to you at the end of the experiment. We will wait for everybody to be finished. Then, we will need between 5 and 10 minutes to prepare the payments. You will be called individually, based on the number of your computer, go into the first room on your right, sign a receipt and receive the earnings in an envelope.

Please note: As every decision can be drawn with the same probability, it is in your interest to take every decision carefully.

For your punctual arrival you receive an additional show up fee of £3.

**PART I**

**Task**

You will see a field on your computer screen, which consists of 100 boxes, as shown on the display.

Boxes are numbered from 1 to 100, starting from the top-left corner.

Your task is to choose how many boxes to collect. So you will be asked to choose a number between 1 and 100.

Boxes will be collected in numerical order as shown on the display.

You earn £0.10 for every box that you collect. Such earnings are only potential, however, because behind one of these boxes hides a bomb and if you collect it, it destroys all earnings accumulated.

You do not know where this bomb lies. You only know that the bomb can be in any place with equal probability. Moreover, even if you collect the bomb, you will not know it until the end of the experiment. At the end of the experiment, the computer will randomly determine, for each of you, the number of the box containing the bomb.

You will collect the bomb if your chosen number is greater than, or equal to, the number of the box containing the bomb.

You will not collect the bomb if your chosen number is smaller than the number of the box containing the bomb.

If you happen to have collected the box where the bomb is located, you will earn nothing. If the bomb is located in a box that you did not collect, you will earn £ 0.10 for each box that you have collected.

When choosing the number of boxes that you would like to collect, you have to write that number twice. You confirm your choice by hitting OK.

After confirming your choice, on your computer screen you will see information about your potential earnings and the field of boxes showing the boxes that you have collected in light grey.

We will start with a practice round. This practice round gives you an opportunity to make sure you understand the types of decisions you make and how these will affect your earnings. After the practice round, the real experiment will start.

Please, press the button OK to go ahead with the experiment.

[***After the practice round, experimenter says aloud:*** *Now the real experiment starts.***]**

**PART II**

Now we start Part II of the experiment.

**[CONTROL]**

In this part, you have to perform again the task performed in Part I.

If you have any questions or problems, please raise your hand and one of the experimenters will come to you and answer your questions in private.

Please, press the button OK to go ahead with the experiment.

**[ANCHORING]**

In this part, you have to perform again the task performed in Part I. Before you do that, you will be first asked about your painting preferences.

We have selected one hundred paintings made by different artists. Paintings are numbered from 1 to 100. You will be shown either two or three of these paintings selected at random and asked to choose which painting you prefer. Your answer will determine which artist you prefer.

After you submit your answer, you will be informed on your preferred artist.

To choose which painting you prefer, click on the button below such painting.

If you have any questions or problems, please raise your hand and one of the experimenters will come to you and answer your questions in private.

Please, press the button OK to go ahead with the experiment.

[***After the choice of the favourite painting***]

Now you have to perform again the task performed in Part I.

If you have any questions or problems, please raise your hand and one of the experimenters will come to you and answer your questions in private.

Please, press the button OK to go ahead with the experiment.

**[RANDOM]**

In this part, you have been randomly matched with two other participants of this experiment. The three of you will form one group in Part II. Groups will remain unchanged for the rest of the experiment.

In this part, you have to perform again the task performed in Part I.

Before and while performing the task you will have information on the choices that you and the other two members of your group made the previous time you performed the task. You will see such information on your screen as shown below:


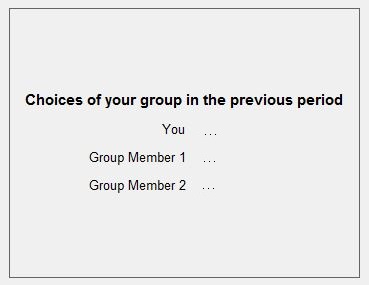


Your payoff will depend only on your own choices.

If you have any questions or problems, please raise your hand and one of the experimenters will come to you and answer your questions in private.

Please, press the button OK to go ahead with the experiment.

**[PAINTING]**

In this part, you have to perform again the task performed in Part I. Before you do that, you will be first allocated in different groups based on your painting preferences.

Everyone will be shown 5 pairs of paintings by two artists. You will be asked to choose which painting in each pair you prefer. Your answers will determine which artist you prefer. You will then be allocated into groups of three individuals based on which artist you prefer. Groups will remain unchanged for the rest of the experiment.

After everyone submits answers, you will be privately informed of the painting preferences of the members of your group.

To choose which painting you prefer, click on either A or B.

If you have any questions or problems, please raise your hand and one of the experimenters will come to you and answer your questions in private.

Please, press the button OK to go ahead with the experiment.

[***After the choices***]

Now you have to perform again the task performed in Part I.

Before and while performing the task you will have information on the choices that you and the other two members of your group made the previous time you performed the task. You will see such information on your screen as shown below:


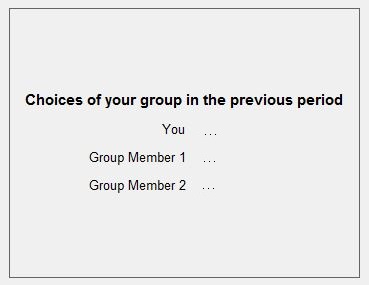


Your payoff will depend only on your own choices.

If you have any questions or problems, please raise your hand and one of the experimenters will come to you and answer your questions in private.

Please, press the button OK to go ahead with the experiment.

**[CHAT]**

In this part, you have to perform again the task performed in Part I. Before you do that, you will be first allocated in different groups and you will do another task that will be explained afterwards.

You will be allocated in different groups based on your painting preferences.

Everyone will be shown 5 pairs of paintings by two artists. You will be asked to choose which painting in each pair you prefer. Your answers will determine which artist you prefer. You will then be allocated into groups of three individuals based on which artist you prefer. Groups will remain unchanged for the rest of the experiment.

After everyone submits answers, you will be privately informed of the painting preferences of the members of your group.

To choose which painting you prefer, click on either A or B.

If you have any questions or problems, please raise your hand and one of the experimenters will come to you and answer your questions in private.

Please, press the button OK to go ahead with the experiment.

[***After the choices***]

Now we will ask you to do a simple task. You will receive two more paintings, one by Klee and one by Kandinsky, and you will have to select the artist who you think made the paintings, respectively.

For each correct answer, you will be rewarded with an additional £1. You will find out your payoff from this task at the end of the experiment.

You can use a group chat program to get help from or offer help to other members in your own group for 5 minutes before answering the questions.

Except for the following restrictions, you can type whatever you want in the lower box of the chat program positioned in the right of your screen. It is enough to press the “Enter” key after you type your message. Messages will be shared only among the members from your own group.

Restrictions on messages:

1. Please do not identify yourself or send any information that could be used to identify you (e.g. age, race, studies, etc…).
2. Please refrain from using obscene or offensive language.

If you have any questions or problems, please raise your hand and one of the experimenters will come to you and answer your questions in private.

Please, press the button OK to go ahead with the experiment.

[***After the group task***]

Now you have to perform again the task performed in Part I.

Before and while performing the task you will have information on the choices that you and the other two members of your group made the previous time you performed the task. You will see such information on your screen as shown below:


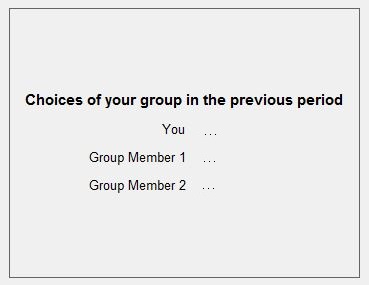


Your payoff will depend only on your own choices.

If you have any questions or problems, please raise your hand and one of the experimenters will come to you and answer your questions in private.

Please, press the button OK to go ahead with the experiment.

**PART III**

Now we start Part III of the experiment. In this part, you have to perform again the box collection task for 10 times.

**[RANDOM, PAINTING AND CHAT]**

The participants you were grouped with are the same for this part of the experiment as well.

Your payoff will depend only on your own choices.

**[ALL]**

As explained before, at the end of the experiment, the computer will randomly choose, for each of you, both the number of the box containing the bomb and one of the twelve decisions of the box collection task that you have taken throughout the three parts of the experiment. Your payment will depend on the number of boxes that you chose to collect in the selected decision.

**[ANCHORING]**

Before each repetition of the box collection task, you will be asked about your painting preferences. You will be shown either two or three paintings picked at random from the one hundred that we have selected and you will have to choose which one you prefer. After you submit your answer, you will be informed on your preferred artist.

**[ALL]**

If you have any questions or problems, please raise your hand and one of the experimenters will come to you and answer your questions in private.

Please, press the button OK to go ahead with the experiment.

[***At the end of the experiment, experimenter says aloud:*** *Now you have to complete a short questionnaire and at the end you will see your earnings displayed on your screen.*]

**[PAINTING and CHAT]**

[***At the end of the questionnaire, experimenter says aloud:*** *Now you can see on the display the paintings made by Klee and the paintings made by Kandinsky.*]

**QUESTIONNAIRE**

1. What is your gender? *Male, Female*
2. What is your ethnicity? *White, Black, Chinese, South Asian, East Asian, Other*
3. What is your age?
4. What is your major?
5. Which year are you in your program? *1,2,3,4,Other*
6. Over the last semester, was the majority of your marks between: *0-39, 40-49, 50-59, 60-69, 70 or more*
7. What is the highest educational qualification of your parents?

|  | Mother | Father |
| --- | --- | --- |
| PhD | ❑ | ❑ |
| Postgraduate Masters degree | ❑ | ❑ |
| Undergraduate degree | ❑ | ❑ |
| Other higher education | ❑ | ❑ |
| A level / Higher or equivalent (secondary education) | ❑ | ❑ |
| GCSE | ❑ | ❑ |
| Other qualification | ❑ | ❑ |
| No qualification | ❑ | ❑ |

1. How many brothers do you have?
2. How many sisters do you have?
3. How often do you drink alcohol? *Never, Once a month or less, 2-4 times a month, 2-4 times a week, Almost every day*
4. Do you smoke? *Yes, No*
5. If yes, how many cigarettes do you smoke in a day?
6. Generally, do you study alone or with someone else? *Alone, With someone else*
7. On average, how many hours a day do you spend with your friends?
8. How do you see yourself: are you generally a person who is fully prepared to take risks or do you try to avoid taking risks? Please choose the value on the scale, where the value 0 means: "risk averse" and the value 10 means: "fully prepared to take risks".
9. People can behave differently in different situations. On a scale where the value 0 means: "risk averse" and the value 10 means: "fully prepared to take risks", how would you rate your willingness to take risks in the following areas? (tick the appropriate)

|  | 0  Risk Averse | 1 | 2 | 3 | 4 | 5 | 6 | 7 | 8 | 9 | 10  Fully prepared to take risks |
| --- | --- | --- | --- | --- | --- | --- | --- | --- | --- | --- | --- |
| While driving or cycling |  |  |  |  |  |  |  |  |  |  |  |
| In financial matters |  |  |  |  |  |  |  |  |  |  |  |
| During leisure and sport |  |  |  |  |  |  |  |  |  |  |  |
| In your occupation |  |  |  |  |  |  |  |  |  |  |  |
| With your health |  |  |  |  |  |  |  |  |  |  |  |
| Your faith in other people |  |  |  |  |  |  |  |  |  |  |  |

**[RANDOM]** You were randomly assigned to a group during the experiment.

**[PAINTING and CHAT]** You were assigned to a group with similar painting preferences during the experiment.

**[RANDOM, PAINTING AND CHAT]** Please answer the following questions by considering a scale with values ranging from 0 (Not at all) to 10 (Very Much).

1. **[CHAT]** How much do you think communicating with your group members helped solve the two extra painting questions?
2. **[PAINTING and CHAT]** How familiar were you with the paintings made by Klee and Kandinsky, respectively, before this experiment?
3. **[RANDOM, PAINTING AND CHAT]** How closely attached did you feel to your own group throughout the experiment?
4. **[RANDOM, PAINTING AND CHAT]** In the repetitions of the task in which you had information about the previous choices of each member of the group, how would you describe the strategies you used?
5. **[RANDOM, PAINTING AND CHAT]** In the repetitions of the task in which you had information about the previous choices of each member of the group, did you consider your relative position in terms of number of boxes previously collected when choosing how many boxes to collect? *Yes, No*
6. **[ANCHORING]** In the repetitions of the task following the choice of your favourite painting, did you consider the numbers of the selected paintings when choosing how many boxes to collect? *Yes, No*
7. How many of the subjects taking the experiment with you in this room do you know?

1. ^^ E-mail address: **f.gioia@sms.ed.ac.uk**. School of Economics, University of Edinburgh, 30 Buccleuch Place, Edinburgh, EH8 9JT, UK. [↑](#footnote-ref-2)
